# Direct Effects of Waterpipe Smoke Extract on Aortic Endothelial Cells: An In Vitro Study

**DOI:** 10.33549/physiolres.935409

**Published:** 2025-02-01

**Authors:** Nur Elena ZAABA, Priya YUVARAJU, Sumaya BEEGAM, Ozaz ELZAKI, Kholoud ARAFAT, Samir ATTOUB, Abderrahim NEMMAR

**Affiliations:** 1 Department of Physiology, College of Medicine and Health Sciences, United Arab Emirates University, Al Ain, United Arab Emirates; 2 Department of Pharmacology and Therapeutics, College of Medicine and Health Sciences, United Arab Emirates University, Al Ain, United Arab Emirates

**Keywords:** Waterpipe smoking, Aortic endothelial cells, Inflammation, Oxidative Stress

## Abstract

Waterpipe smoking (WPS) has adverse health effects that include endothelial dysfunction with mechanisms involving oxidative stress and inflammation. Nonetheless, there is a scarcity of data on the direct impact of WPS on endothelial function. In this study, we assessed the *in vitro* effects of waterpipe smoke extract (WPSE) on aortic endothelial cell lines, namely the TeloHAEC. The WPSE markedly caused concentration- and time-dependent decreases in cellular viability. When compared with the control, at a concentration of 20 % and an incubation period of 48 h, the WPSE significantly increased the levels of lactate dehydrogenase, and markers of oxidative stress including thiobarbituric acid reactive substances, superoxide dismutase, catalase, and reduced glutathione. Moreover, the concentrations of proinflammatory cytokine (tumor necrosis factor α), and adhesion molecules (E-selectin and intercellular adhesion molecule-1) were also significantly augmented. Likewise, WPSE triggered mitochondrial dysfunction, DNA oxidative damage, as well as apoptosis in TeloHAEC cells. Similarly, cells cultured with WPSE have shown increased expression of phosphorylated nuclear factor-κB and hypoxia-inducible factor 1-α (HIF-1α). In conclusion, our study showed that WPSE triggers endothelial inflammation, oxidative stress, DNA damage, mitochondrial dysfunction, and apoptosis *via* mechanisms involving the activation of nuclear factor-κB and HIF-1α.

## Introduction

Waterpipe smoking (WPS), also known as hookah or shisha is a growing phenomenon worldwide, particularly among young adults and women, and has become a growing public health issue [1]. Several factors are attributed to WPS’s popularity, including the use of fruit-flavored tobacco, which produces pleasant fruity aromas and tastes [2]. Moreover, the rise in popularity of WPS can also be ascribed to the misconception that the fruity and cool smoke inhaled is less hazardous and less addictive compared to cigarette smoking (CS) [3,4]. However, there is now a substantial body of evidence to debunk these misconceptions and link WPS to adverse health effects, with a particular emphasis on its effect on endothelial and cardiovascular health [5].

Waterpipe (WP) smoke is a complex concoction of various toxic elements, comprising carbon monoxide, nicotine, heavy metals, polycyclic and heterocyclic aromatic hydrocarbons, volatile organic compounds, and particulate matter [1,5]. These toxic compounds are the products of the combustion of heated tobacco and from the burning of the charcoal used to heat the tobacco. Previous epidemiological and clinical studies have shown that contrary to popular belief WPS is in fact more harmful than CS [6]. It has been reported that one session of WPS could produce up to 6.5 times the amount of carbon monoxide, 1.7 times more nicotine, and 46 times more tar relative to a session of a single cigarette [6]. This is probably due to the longer smoking session and deeper inhalation compared to CS [6].

Human telomerase reverse transcriptase-immortalized aortic endothelial cells (TeloHAEC) are cells that demonstrate the characteristics of endothelial cells in the aorta and heart. The endothelial cells play various roles in cardiovascular homeostasis, as they regulate the vascular tone, thrombosis, angiogenesis, and inflammation [7]. Moreover, the endothelial cells are the second most exposed cells after the alveolar cells, and are known to be vulnerable to the deleterious effects caused by the products of tobacco combustion [8]. In response to cardiovascular risk factors, including WPS, the endothelial cells release large amounts of vasoactive substances and inflammatory mediators [8]. This could potentially alter normal endothelial functions and lead to vascular endothelial dysfunction, which precedes the development of atherosclerosis and coronary heart disease [8].

The adverse *in vivo* effects of WPS on endothelial and cardiovascular health have been previously described [9–11]. We have recently demonstrated that WPS inhalation triggers lung injury and endothelial inflammation, oxidative stress, and apoptosis, which were associated with nuclear factor-κB activation and SIRT1 down-regulation [10]. These findings led us to speculate whether these effects were due to the spill-over of inflammatory mediators and pro-oxidants from the injured lungs into the circulatory system, or whether they were a result of direct contact between WP smoke toxicants crossing the alveolar-capillary barrier and interacting directly with vascular endothelium. Therefore, in order to explore this aspect, it is essential that we shift our focus from *in vivo* mainstream WPS to study the effect of waterpipe smoke extract (WPSE) on endothelial cells, which are the focal cells of the vascular system.

Consequently, the objective of our study is to evaluate the possible direct impact of WPSE on aortic endothelial cells *in vitro* and the underlying mechanisms involved by assessing various relevant parameters including markers of oxidative stress, inflammation, mitochondrial dysfunction, DNA oxidative damage, and apoptosis.

## Material and Methods

### Setup and preparation of waterpipe smoke extract

**Fig. 1:**
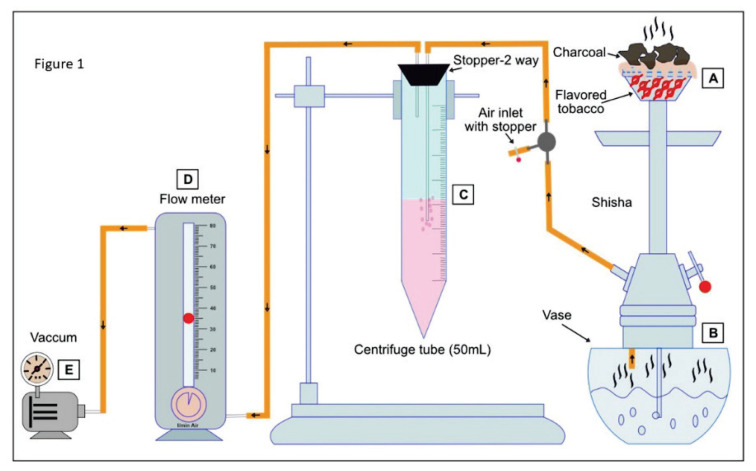
The schematic diagram represents the preparation of waterpipe smoke extract (WPSE) from flavored tobacco smoke in the shisha set-up. The smoke produced by burning flavored tobacco over charcoal (**A**). The distilled water in the glass vase functions as a conduit for the tobacco smoke (**B**). The tobacco smoke movement into a 10 ml serum-free EBM™-2 Clonetics® media is shown by an arrow in the pipeline (**C**). The smoke was drawn at a constant rate of 35 ml/min using a flow meter (**D**). Negative pressure was regulated by vacuum suction (**E**).

Figure 1 depicts a modified version of a previously described set up pertaining with the preparation of WPSE [2]. Ten grams of apple-flavored tobacco purchased from Al Fakher Tobacco Trading (Ajman, UAE) were placed in a shisha bowl, covered with pierced aluminum foil, and topped with red hot charcoal to create waterpipe smoke extract [2]. The smoke was channeled through distilled water in a glass vase. Tobacco smoke from the waterpipe was collected into 10 ml of serum-free endothelial basal medium (EBM™)-2 media from Lonza (Walkersville, MD, USA) using vacuum suction in a 50 ml centrifuge tube at a flow rate of 35 ml/min. The optical density of the WPSE obtained was systematically measured each time before the exposure of WPSE to TeloHAEC cells in order to ensure that the nicotine content was consistent with that of a regular WPS session. A delta OD of 3±0.2 was found to be equivalent to 100 % WPSE when measured at 320 and 540 nm [12,13]. The resulting WPSE was used further to study the cell viability on TeloHAEC cells.

### Cell culture and reagents

The TeloHAEC (hTERT-immortalized human aortic endothelial cells) were maintained in EMB-2 medium kit (CC-3162, Lonza, Walkersville, MD, USA) at 37 °C in a 5 % CO_2_ atmosphere. The WPSE was diluted in full medium until it reached the desired concentration of 20 %.

### Cell viability

TeloHAEC cells were seeded into 96-well plates at a density of 9000 cells/well (n=4 independent experiments). After 24 h [14], cells were incubated with WPSE at 1, 5, 10 and 20 % in duplicate for 1, 6, 24 and 48 h while control cells were incubated in media without WPSE. A CellTiter-Glo® Luminescent Cell Viability assay from Promega Corporation (Madison, WI, USA) was used at the indicated time points to determine the effects of WPSE on cellular viability by quantifying the adenosine triphosphate (ATP) that is proportional to the number of metabolically active cells. The luminescence was evaluated with a GloMax® Luminometer by Promega Corporation (Madison, WI, USA). By comparing WPSE-treated cells to control cells, where viability was arbitrarily set at 100 %, we were able to express cellular viability as a percentage (%).

### Biochemical markers

TeloHAEC cells were seeded into 12-well plates at a density of 80000 cells/well (n=4 independent experiments). After 24 h, the cells were incubated with WPSE at a concentration of 20 % in 1 ml of culture media per well for 48 h while control cells were incubated in 1 ml of the same media without WPSE. Supernatants of the culture media were then collected and analyzed for several biochemical markers.

### Lactate dehydrogenase (LDH)

The release of LDH was evaluated as a surrogate marker of cell damage using a modified assay as previously described [15]. Briefly, in a 96 well plate, 50 μl of assay reagent (made by combining 1-methoxyphenazine methosulfate, iodonitrotetrazolium chloride, and β-nicotin-amide adenine dinucleotide sodium salt a few minutes before starting the assay) was added to a 50 μl cell culture media supernatant. After a quick shake on an orbital shaker, the plate was left to incubate for 60 min at room temperature in the dark. Finally, 50 μl of 1 M acetic acid was added to each well to terminate the reaction and stabilize it. A plate reader was used to measure the absorbance at 490 nm.

### Thiobarbituric acid reactive substances (TBARS), superoxide dismutase (SOD), catalase and reduced glutathione (GSH)

Cayman Chemicals kits (Ann Arbor, MI, USA) were used to measure TBARS, SOD and catalase activities. Whereas the concentration of GSH was measured using a kit purchased from Sigma Aldrich Co. (St. Louis, MO, USA) [16].

### Tumor necrosis factor α (TNF α), E-selectin and intercellular adhesion molecule-1 (ICAM-1)

The concentrations of TNF α, E-selectin and ICAM-1 were assessed using ELISA kits purchased from R&D Systems (Minneapolis, MN, USA).

### Mitochondrial membrane potential analysis with JC-1 probe

A kit from Cayman Chemicals (Ann Arbor, MI, USA) was used to perform the JC-1 mitochondrial membrane potential assay.

### DNA oxidative damage

The level of 8-hydroxy-2-deoxyguanosine (8-OH-dG) was measured using an ELISA kit from Cayman Chemicals (Ann Arbor, MI, USA).

### Cytochrome C and cleaved caspase 3

Cytochrome C and cleaved caspase 3 were quantified using ELISA kits from R&D Systems (Minneapolis, MN, USA).

### Phosphorylated nuclear factor κB (Phospho-NF-κB) and Hypoxia-inducible factor 1-α (HIF-1α)

The concentration of phospho-NF-κB was measured using a kit from Cell Signalling Technology (Danvers, MA, USA), whereas the concentration of HIF-1α was determined by using an ELISA kit from R&D Systems (Minneapolis, MN, USA).

## Results

### Cell viability

**Fig. 2:**
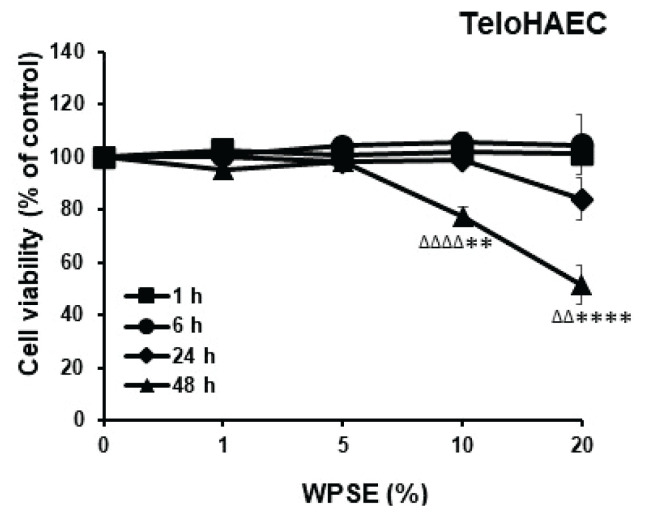
Cell viability assay for TeloHAEC cells after 1, 6, 24 and 48 h of incubation with waterpipe smoke extract (WPSE) at different concentrations (0, 1, 5, 10 and 20 %). Cellular viability is significantly reduced after 48 h incubation with 20 % WPSE. Mean ± SEM (at least 3 independent experiments). Statistical analysis by one-way analysis of variance followed by Holm-Sidak’s test. ^ΔΔ^ *p*<0.01 group incubated with 20 % WPSE for 48 h compared with the group incubated with 0 % WPSE for 1 h and ^ΔΔΔΔ^ *p*<0.0001 group incubated with 10 % WPSE for 48 h compared with the group incubated with 0 % WPSE for 1 h. ** *p*<0.01 group incubated with 10 % WPSE for 48 h compared with the group treated with 0 % WPSE for 48 h and **** *p*<0.0001 group incubated with 20 % WPSE for 48 h compared with the group treated with 0 % WPSE for 48 h.

The effect of the increasing concentration of WPSE was investigated on the TeloHAEC cell lines. As shown in Figure 2, the WPSE significantly reduced the viability of TeloHAEC in concentration- and time-dependent manner. The half-maximal inhibitory concentration (IC_50_) of WPSE at 48 h was approximately at 20 % of WPSE, hence cells incubated with this concentration and at this duration was selected to represent the WPSE group for the subsequent analyses.

### LDH

**Fig. 3:**
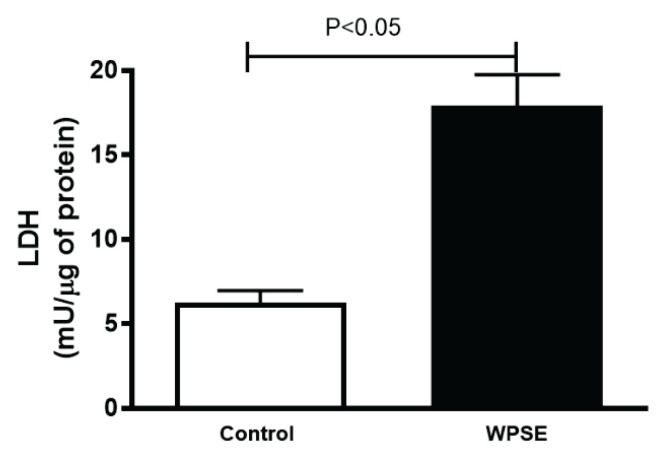
Lactate dehydrogenase (LDH) released in the culture media of TeloHAEC cells incubated with 20 % waterpipe smoke extract (WPSE) and in media with 0 % WPSE for 48 h. Mean ± SEM (n=4 independent experiments). Statistical analysis by one-way analysis of variance followed by Holm-Sidak’s test.

The cytotoxic effects of WPSE on TeloHAEC cell lines were evaluated using the LDH assay. Figure 3 shows that when compared with the control group, cells incubated in 20 % WPSE displayed a significant increase in the level of LDH (*p*<0.05).

### TBARS, catalase, SOD, GSH

**Fig. 4:**
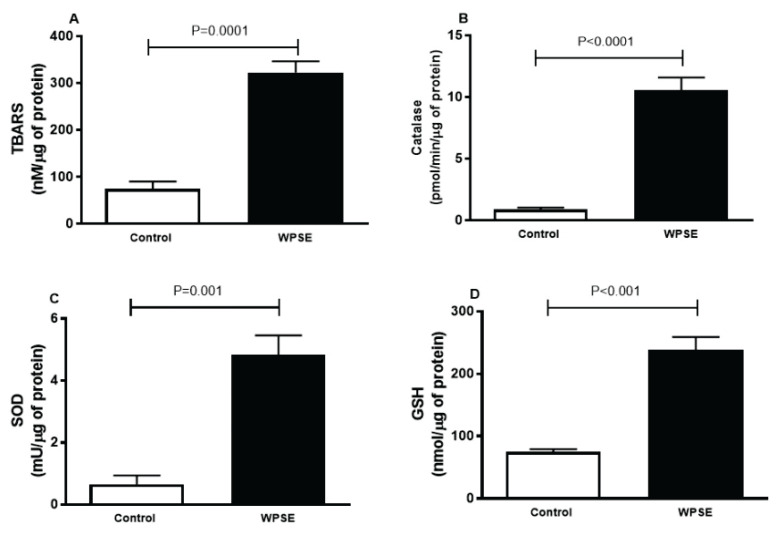
Thiobarbituric acid reactive substances (TBARS, **A**), catalase (**B**), superoxide dismutase (SOD, **C**) and reduced glutathione (GSH, **D**) released in the culture media of TeloHAEC cells incubated with 20 % waterpipe smoke extract (WPSE) and in media with 0 % WPSE for 48 h. Mean ± SEM (n=4 independent experiments). Statistical analysis by one-way analysis of variance followed by Holm-Sidak’s test.

In order to evaluate the oxidative stress of cells incubated with WPSE versus the control, we have quantified the marker of lipid peroxidation, TBARS. As depicted in Figure 4, the level of TBARS was significantly elevated (*p*=0.0001) in cells exposed to 20 % WPSE, compared with the control group. Likewise, a significant increase in catalase (*p*<0.0001) and SOD (*p*=0.001) activities, and GSH (*p*<0.001) concentration were observed in WPSE-treated cells compared with the control group.

### TNF α, E-selectin, ICAM-1, mitochondrial membrane potential analysis with JC-1 probe and DNA oxidative damage

**Fig. 5:**
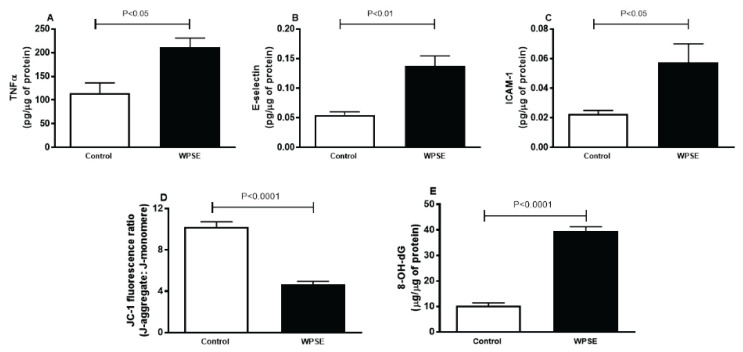
Tumor necrosis factor α (TNF α, **A**), E-selectin (**B**), intercellular adhesion molecule-1 (ICAM-1, **C**), JC-1 mitochondrial membrane potential (**D**) and 8-hydroxy-2-deoxyguanosine (8-OH-dG, **E**) released in the culture media of TeloHAEC cells incubated with 20 % waterpipe smoke extract (WPSE) and in media with 0 % WPSE for 48 h. Mean ± SEM (n=4 independent experiments). Statistical analysis by one-way analysis of variance followed by Holm-Sidak’s test.

Figure 5A-C shows the concentrations of the TNF α, as well as the adhesion molecules E-selectin and ICAM-1 in TeloHAEC cells incubated with WPSE compared with the control group. The concentrations of TNF α (*p*<0.05), E-selectin (*p*<0.01) and ICAM-1 (*p*<0.05) were significantly increased in WPSE group compared with the control.

Figure 5D depicts that WPSE group has significantly lower JC-1 fluorescence ratio (*p*<0.0001) compared with the control group.

When compared with the control group, cells incubated with 20 % WPSE released significantly higher level of the oxidized derivative of deoxyguanosine 8-OH-dG (*p*<0.0001, Fig. 5E), a major product of DNA oxidation.

### Cytochrome C, cleaved caspase 3, phospho-NF-κB and HIF-1α

**Fig. 6:**
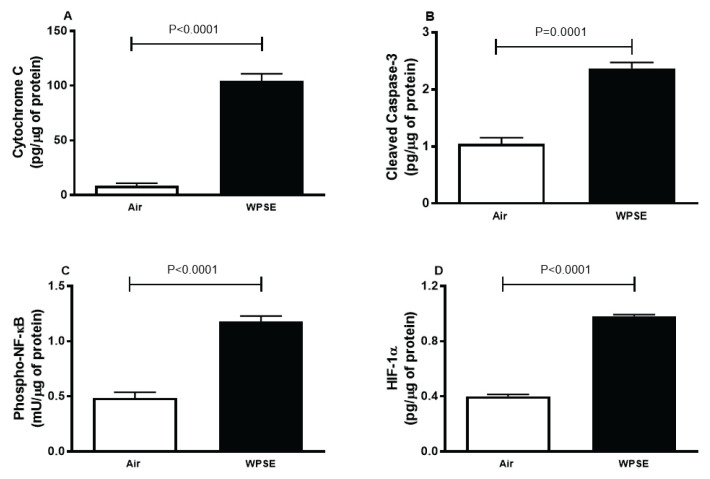
Cytochrome C (**A**), cleaved caspase 3 (**B**), phosphorylated nuclear factor-κB (phospho-NF-κB, **C**) and hypoxia-inducible factor 1-α (HIF-1α, **D**) released in the culture media of TeloHAEC cells incubated with 20 % waterpipe smoke extract (WPSE) and in media with 0 % WPSE for 48 h. Mean ± SEM (n=4 independent experiments). Statistical analysis by one-way analysis of variance followed by Holm-Sidak’s test.

Figure 6A–B shows that there was a significant increase in the levels of cytochrome C (*p*<0.0001) and cleaved caspase 3 (*p*=0.0001) in WPSE group compared with the control.

Figure 6C shows that cells incubated with 20 % WPSE had a significantly higher level of phospho-NF-κB compared with the control (*p*<0.0001).

Figure 6D depicts that the concentration of HIF-1α was remarkably increased in WPSE-exposed cells, compared with the control group (*p*<0.0001).

## Discussion

The present study has provided experimental evidence of the adverse effects of the *in vitro* exposure to WPSE on the aortic endothelial cells involving mechanisms including oxidative stress, inflammation, DNA damage and apoptosis, all of which are crucial in the development and the progression of endothelial dysfunction.

Both epidemiological and experimental studies have shown that WPS is associated with the deterioration of the vascular endothelium. This significantly increases the risk of endothelial dysfunction which precedes various clinical manifestation of coronary artery diseases including atherosclerosis, thrombosis, and sudden cardiac death [10]. The adverse vascular endothelial effects associated with WPS can be ascribed to the intricate interplay between oxidative stress and inflammation [17]. These mechanisms alter the normal endothelial functions, and lead to several adverse effects such as vasoconstriction, thrombosis and apoptosis which are the precursors of cardiovascular diseases [18]. It has been hypothesized that inhaled toxicants induces oxidative stress and inflammation either by direct contact with the endothelium, or indirectly where the toxicants cause oxidative stress and inflammation locally in the lungs which can lead to the release of proinflammatory cytokines and pro-oxidants which can cross the alveolar capillary barrier and reach the systemic circulation [19–21].

We have previously elucidated the adverse *in vivo* endothelial effects of one month exposure to WPS in mice, where WPS induced lung injury and increased the aortic concentrations of proinflammatory cytokines as well as markers of oxidative stress [10]. Moreover, the same observation was found in the heart of mice, along with an increase in prothrombic events [22]. This information led us to speculate whether these effects were due to the spill-over of pro-oxidants and inflammatory mediators from the lungs to the circulatory system following WPS inhalation, or perhaps were the results of a direct contact between the WP smoke toxicants with the vascular endothelium. The present study was designed to provide essential information on the direct impact of WPSE on vascular endothelium particularly on the aortic endothelial cells.

In this study, TeloHAEC endothelial cells from human aorta were incubated in media containing WPSE at the concentration of 20 % for the duration of 48 h. Prior to that, we have carried out the cell viability assay to determine the half-maximal inhibitory concentration of the WPSE, and found that at 20 % of concentration and 48 h of incubation period the extract has shown maximum efficiency by inhibiting the biological processes of half of the population of cells, hence, selected as the most suitable concentration and time point [23]. We subsequently assessed the level of LDH, a biomarker of cytotoxicity which could indicate endothelial cell damage and found to be elevated in various diseases, including coronary artery disease [24]. In fact the measure of LDH in cell culture medium could provide accurate measurement of cell viability [15]. Presently, we found that the concentration of LDH was significantly higher in cells treated with WPSE compared to the control.

In light of the latter observations, we wanted to investigate the involvement of oxidative stress in the present endothelial cytotoxicity by assessing several markers of oxidative stress including TBARS, one of the many reactive aldehydes produced as a result of lipid peroxidation [25–28]. We found that the concentration of TBARS was significantly elevated in the WPSE group compared with the control group, an observation that was confirmed by a previous study that delineates the effect of cigarette smoke extract (CSE) on human bronchial epithelial cells [29]. Similarly, the activities of SOD and catalase and the concentration of GSH were significantly augmented in cells exposed to WPSE compared with the control group. The consistent increase in all antioxidant parameters is possibly due to the compensatory mechanism to counterbalance the oxidative stress environment, during which the cells produce more antioxidants to neutralize the reactive oxygen species (ROS) induced by the toxicants in WP smoke [25]. These observations suggest that the vascular oxidative stress caused by WP smoke is probably due to the ability of WP smoke toxicants to directly induce oxidative stress in the endothelium. Likewise, the concentration of the proinflammatory cytokine TNF α was significantly higher in cells exposed to WPSE compared with the control group, suggesting that in addition to oxidative stress, inflammation could also be directly induced by WP smoke toxicants. These findings corroborate with previous clinical and experimental studies where WPS has been reported to cause systemic and vascular oxidative stress and inflammation [5,8,10].

During inflammation, the release of proinflammatory cytokines in the inflamed site induces the expression of endothelial-cell specific adhesion proteins, particularly E-selectin and ICAM-1 [30]. These important markers of endothelial disorder are responsible in recruiting and transporting leukocyte to the site of inflammation [31]. Here, we found that the concentrations of E-selectin and ICAM-1 were significantly elevated in the WPSE group, which is consistent with our previous in vivo findings in the aortic tissue and plasma of mice exposed to WPS [10,11].

Several studies have reported that the toxicant in tobacco smoke could directly alter the mitochondrial DNA and inhibit mitochondrial proteins in cardiac and vascular tissues in mice and rabbits [32]. This subsequently leads to the reduction of the mitochondrial membrane potential [33]. Our findings are in agreement with these reports, as we found a significant reduction in mitochondrial membrane potential in the aortic endothelial cells exposed to WPSE compared with the control. The mitochondrial membrane potential plays a pivotal role in oxidative phosphorylation, the latter forms transmembrane potential of hydrogen ion, an essential component for ATP production [34]. Subse-quently, the reduction of the mitochondrial membrane potential leads to the reduction of ATP produced and the increase of free radical production, which could exacerbate the oxidative stress and inflammation, and consequently cause damage to cellular components, including DNA [25,35]. In the present study, we have evaluated the level of 8-OH-dG, one of the most predominant oxidative lesions caused by ROS, that affect both nuclear and mitochondrial DNA [36], and found that the concentration of this marker was significantly elevated in WPSE group compared with the control group. The presence of DNA damage could trigger both cell arrest and DNA repair mechanisms. However, some of the oxidative DNA damage might not be properly repaired due to the severity of the damage and subsequently could lead to apoptosis, a programmed cell death [37].

It is well established that the mitochondrial-induced apoptosis occurs intrinsically and initiated upon the release of cytochrome C into the cytoplasm as a result of cellular oxidative stress or damage [38]. Cytochrome C then, recruits and activates caspase 9, which in turn partakes in the cleavage and the activation of caspase 3, an enzyme that is critical in the execution of apoptosis [38]. In addition to that, the extrinsic pathway led by TNF α could initiate apoptosis as well [39]. Presently, we found that WPSE group expressed significantly higher levels of cytochrome C and cleaved caspase 3, compared with the control group. These observations are in line with our previous *in vivo* observations which showed that WPS induces oxidative stress leading to the elevation of cytochrome C and cleaved caspase 3 activity in heart and aortic tissues, suggesting mitochondrial damage and the initiation of apoptosis [10]. The elevated concentrations of TNF α and cytochrome C in this study suggests that it is possible that apoptosis was initiated *via* both intrinsic and extrinsic pathways, which converge on caspase 3 activation and subsequently led to the increase of its level [40]. These findings corroborate the previously reported data, including our own, where we have elucidated the apoptotic effects of WPS in the aorta of mice [10]. Moreover, when exposed to CSE, overexpression of caspase 3 was observed in human umbilical venous endothelial cells [41]. While another study showed that CSE caused DNA-strand breaks and induced the activation of the pro-apoptotic marker P53, which resulted in apoptosis, as well as mitochondrial membrane depolarization [42].

To gain a deeper insight into the mechanisms responsible for the adverse effects of WPS, we have evaluated the levels of phospho-NF-κB. Here, we found that phospho-NF-κB was markedly elevated in WPSE group compared with the control group, which concurred with previous reports [10,43]. In human epithelial cells, it has been reported that CSE induced the activation of the NF-κB [43]. Moreover, we have previously reported that in mice, WPS caused significant elevation in the level of phospho-NF-κB in the aorta [10]. This suggests that the initiation of inflammation might be, at least in part, due to the activation of the NF-κB.

Furthermore, we have evaluated the concentration of HIF-1α. The latter is an important marker for hypoxia induced by oxygen deprivation which can be triggered by various external factors including cigarette smoking [44]. During hypoxia, the expression of HIF-1α is heavily regulated by the NF-κB pathway [45]. Previous study has shown the interdependent relationship between HIF-1α and NF-κB, using luciferase under the control of NF-κB-expressing transgenic mice to demonstrate that hypoxia activates NF-κB in the heart and lungs at normobaric atmosphere [45]. Moreover, the same study reported the suppression of hypoxia-induced HIF-1α in cells in which the canonical NF-κB pathway has been inhibited [45]. These findings suggest that HIF-1α plays an important role in inflammation. Additionally, several studies have indicated that the mitochondrial ROS alone can activate HIF-1α, while others suggest that in addition to NF-κB, ROS and the MAPK/AKT signalling could modulate the HIF-1α activation [44]. In the present study, the concentration of HIF-1α was significantly elevated in WPSE group compared with the control. The aforementioned observation coincides with a previous study on CSE on alveolar and bronchial epithelium-derived cells, where the level of HIF-1α increased in a ROS-dependent manner which subsequently led to inflammation and apoptosis [44].

In conclusion, the aortic endothelial cells exposed to 20 % WPSE for 48 h exhibited oxidative stress, inflammation, mitochondrial dysfunction which subsequently led to DNA damage and apoptosis. These findings suggest that the toxicants in the WPSE could potentially cross the alveolar-capillary barrier and directly cause oxidative stress and inflammation in the endothelial cells. However, further work is warranted in order to determine which toxicants are specifically responsible for the adverse effects, and the mechanisms involved in this action.
